# Patients' and families' opinions via suggestion boxes for improving hospital care for cancer patients: Analysis of 3419 cases over 11 years

**DOI:** 10.1002/cnr2.1509

**Published:** 2021-07-15

**Authors:** Kenji Takao, Tetsuo Nishimura, Kenta Yasui, Asami Nakagawa, Ken Yamaguchi

**Affiliations:** ^1^ Osaka University Graduate School of Medicine Program of Multi‐professional Team Approach at Shizuoka Cancer Center, Division of Health Sciences Suita‐city Osaka Japan; ^2^ Risk Management & Quality Control Office Shizuoka Cancer Center Hospital Sunto‐gun Shizuoka Japan; ^3^ Patient Support & Inquiries Shizuoka Cancer Center Hospital Sunto‐gun Shizuoka Japan; ^4^ Shizuoka Cancer Center Sunto‐gun Shizuoka Japan

**Keywords:** patient experience, patient‐centered, patients' complaints, quality improvement, suggestion box

## Abstract

**Background:**

There has been a paradigm shift in cancer treatment from curing disease to both curing disease and caring for patients. In terms of care for patients, the opinions of patients and their families are important for improving medical services.

**Aim:**

The opinions of patients and families were collected at Shizuoka Cancer Center (SCC) and examined from the standpoint of characteristics, response status, and temporal changes.

**Methods:**

Patients' and families' opinions submitted to suggestion boxes at SCC over an 11‐year period (2005–2015) were analyzed. Opinions were categorized as complaints or compliments, with sub‐categories including “facilities, goods, and medical care,” “people,” “time,” and “other.” The status of facilities' response to complaints was categorized as “responded,” “did not respond”, and “difficult to respond”.

**Results:**

Changes in the number of opinions and content over time were examined. In total, 3419 opinions were collected; 69.1% were complaints, and 30.5% were compliments. Of the complaints, 53.4% were related to “facilities, goods, and medical care” (mainly focusing on “poor product quality” and “shortage of goods”), 38.7% were related to “people,” and 7.7% to “time.” Of the compliments, 82.4% were about “people,” all of which concerned facility staff. Facilities' responses to complaints were as follows: “responded” (42.4%), “did not respond” (14.3%), and “difficult to respond” (43.3%).

**Conclusion:**

Understanding patients' and families' opinions is effective for strengthening trust between patients and healthcare professionals, promoting holistic care.

## 1 INTRODUCTION

The Institute of Medicine (IOM) recommends a patient‐centered approach as a goal for the medical system.^1^ Evaluation by patients, as the recipients of medical care, and incorporation of patients' perspectives are increasingly important.^2^, ^3^, ^4^ In Japan, 1 in 2 people will get cancer during their lives, and 1 in 3 people will die from cancer.^5^ There has been a paradigm shift in cancer treatment from medical treatment for curing disease to medical care that focuses on both curing and caring for patients. The latter requires individual care for cancer patients as well as the treatment of cancer lesions. Thus, medical staff are required to provide holistic medical care.

**FIGURE 1:**
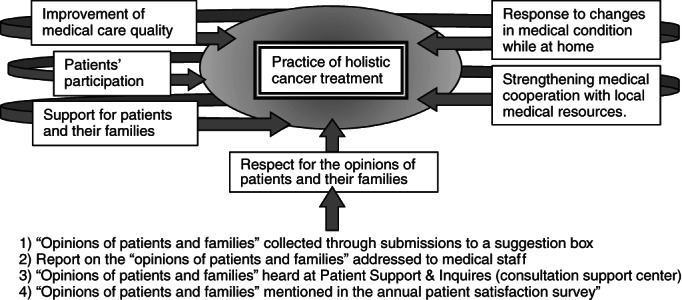
Implementation of holistic cancer treatment. The importance of respecting the opinions of patients and their families, and other factors

Important factors for practicing holistic medical care for cancer patients include improvement of medical care quality, patients' participation, support for patients and their families, responding to changes in patients' medical condition while at home, and strengthening medical cooperation with local medical resources. Additionally, respecting the opinions of patients and their families is considered equally important (Figure 1). Trust between the patient (and their family) and all healthcare professionals is required as the foundation for all of these factors.

Since its establishment in 2002, Shizuoka Cancer Center (SCC) has worked to strengthen trust between patients (and their families) and healthcare professionals, to provide holistic cancer care. Based on experience in cultivating trust between various parties, SCC has recognized the importance of considering the opinions of patients and their families. Thus, SCC currently collects such opinions using four methods: (1) opinions of patients and families collected through submissions to a suggestion box; (2) reports on the opinions of patients and families conveyed to medical staff; (3) opinions of patients and families heard at Patient Support & Inquires (a consultation support center); and (4) opinions of patients and families collected in an annual patient satisfaction survey. Patients' voices are an important source of information for improving the quality of medical care, with a shift from “disease‐centered” to “patient‐centered” approaches.^6^ SCC responds to the submissions received where possible, and communicates the status of the facilities' responses to the person who shared the opinion.

The current study examined patients' and families' opinions collected via suggestion boxes at SCC over an 11‐year period using the four aforementioned measures.

The characteristics of these opinions, the facilities' response status, and temporal changes in opinions were investigated. The results contribute to an assessment of the overall usefulness of opinions for medical institutions. A “submission” is a patient's action to provide their comments to the hospital. The contents of a submission are processed by classification and are re‐written by researchers into a short message for further analysis as an “opinion.”

## 2 METHODS

### 2.1 Data

SCC collects opinions of patients and families using four methods: submissions submitted to suggestion boxes (placed in 14 locations in the hospital), reports from medical staff, conversations at Patient Support & Inquires, and an annual patient satisfaction survey. Opinions of patients and families are collected by SCC's Risk Management & Quality Control Offices, where the content of each opinion is investigated and carefully assessed to ascertain its validity. The correlation of opinions with the policies of SCC and past responses are then confirmed at the facilities' executive councils to determine the final response to be given. Past submissions and responses are stored in a database and can be easily retrieved. Using these, the draft responses are created. The final response decision is made at the weekly SCC Supreme Decision Meeting.

The basic response policy consists of three points: (1) to respond when an opinion is justified and responding to it is feasible; (2) to not respond when no institutional response is required; and (3) to mark the opinion as “difficult to respond” if the opinion is justified but it is difficult to make an improvement or to respond because the relevant details are unknown. Thereafter, for patients (and/or their families) who have submitted their opinions and requested a response, the results of the investigation, along with SCC's response policy, are communicated via a meeting or in writing. In cases where opinion‐givers may have wished not to be notified of responses or to remain anonymous, SCC discloses the opinion and response status in a booklet titled “*A Collection of Opinions*,” which visitors including patients and their families can view.

In this study, the SCC booklet, “A Collection of Opinions,” which covers an 11‐year period (2005–2015), was examined. This booklet excludes complaints with names attached, to which SCC provided individual responses. Using these data, response status and temporal changes in opinions were examined, as well as the usefulness of submissions for hospital management. The SCC booklet, “A Collection of Opinions,” consists of submissions from anonymous contributors and those who did not request a direct response. Submissions from contributors who requested direct feedback are not included in this booklet because they have already received a response. The feedback system is introduced in a video hospital guide for patients and their families at their first visit. It is also described in the booklet handed to patients at the time of outpatient visits and hospitalization. SCC staff members in the RM/QC collect the submissions from suggestion boxes in 14 locations (e.g., in front of the elevator, in the information corner of each ward day room, next to the telephone near the front entrance, in the in‐hospital library, at the outpatient medical accounting counter, and in the family waiting room).

### 2.2 Analysis method

Taxonomies are commonly used to understand collected patient opinions. Classification is used to analyze causes, make judgments about appropriate responses, share within the organization, and improve the efficiency of responses.^7^ A highly effective taxonomy (three areas, seven categories, 26 subcategories) was proposed in a systematic review.^8^ However, because opinions such as patient complaints in medical care may be influenced by the medical system and individuals,^9^ it is necessary to avoid using taxonomies developed in other countries, with unique patient opinion analysis systems in each country. Many taxonomies in Japan have been created independently,^10^, ^11^, ^12^ with no widely established taxonomy. In the current study, the opinions of patients and families in “*A Collection of Opinions*” between 2005 and 2015 were categorized as follows:

1. First, one author independently read 1101 submissions in “A Collection of Opinions” from a 3‐year period, divided them into groups based on the contents, and conducted a short sentences.
2. The contents of the opinions were categorized as “complaints,” “compliments,” or “other.” For “complaints,” along with the expressed dissatisfaction of patients and families, opinion‐givers' requests and proposals were also included. “Compliments” consisted of opinions indicating satisfaction and/or gratitude. “Complaints” and “compliments” require different responses from hospitals. Many international studies have classified “complaints” as negative and “compliments” as positive.^13^, ^14^, ^15^ However, from the perspective of customer service and medical service, “complaints” are not a cause of harm, but rather an opportunity for insight.^7^, ^16^
3. The contents of “complaints” and “compliments” opinions were then further classified into “facilities, goods, and medical care,” “people,” “time,” and “other.” “Facilities, goods, and medical care” included facilities, equipment, consumable goods, and hospital systems related to patient care. “People” was divided into “institution staff” and “non‐institution individuals”. Institution staff included medical staff (e.g., doctors, nurses, and co‐medical staff) but also administrative staff working at various counters and facility operations staff (e.g., staff who aided with meals, cleaning, and shops). “Non‐institution individuals” included patients, their families, and other facility users. “Time” covered time‐related concepts, such as waiting time.
4. Regarding the contents of opinions, the responses of facilities to complaints were divided into “responded” (i.e., the opinion is justified, and a response is feasible), “did not respond” (i.e., there is no need for the institution to respond), or “difficult to respond” (i.e., the status quo had to be maintained as per past correspondence, or the details were unclear).
5. Two researchers familiar with cancer medicine evaluated the appropriateness of the classification and examined the validity of the classified opinions. If there were disagreements, the three parties examined and discussed the classifications until they agreed, and tried to ensure their validity.
6. The same author as in step 1 continued reading and classifying the remaining 8 years of submissions from “A Collection of Opinions” following the procedure of steps 1–5.

## 3 RESULTS

### 3.1 Number of submissions and opinions

**FIGURE 2:**
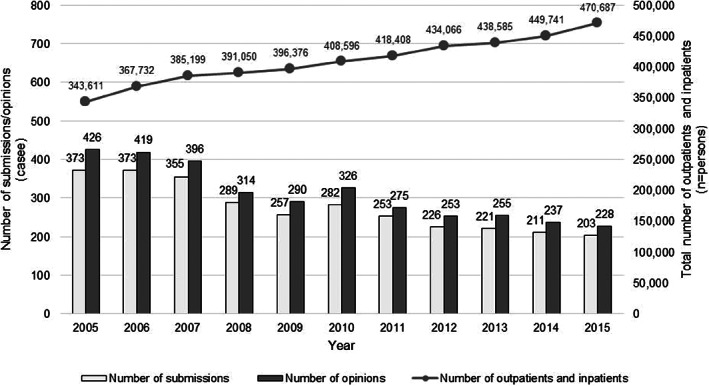
Total number of outpatients and inpatients, submissions, and opinions

During the 11‐year period (2005–2015), the total number of submissions published in “*A Collection of Opinions*” was 3043 (277 submissions/year on average), with a total of 3419 opinions (311/year on average) submitted to suggestion boxes. While the total number of out‐ and in‐patients at SCC increased annually from 343 611 in 2005 to 470 687 in 2015, the number of submissions/opinions submitted to suggestion boxes was highest, at 373/426, respectively, in 2005, while 2015 recorded the lowest number of submissions/opinions at 203/228, respectively. Although there were some fluctuations during this period, overall, submissions/opinions decreased annually (Figure 2). The ratio of the number of opinions based on the total number of out‐ and in‐patients halved during the 11‐year period. Additionally, during the 11 years surveyed in this study, there were no system changes that could affect the percentage of opinions in the SCC.

### 3.2 Breakdown of categories

Regarding the content categories of patients' and families' opinions over 11 years, there were 2363 (69.1%) complaints, 1042 (30.5%) compliments, and 14 (0.4%) classified as “other.” Complaints were predominantly related to “hospital food,” “equipment,” “response and attitude,” and “waiting time.” Most compliments referred to “environment,” “equipment,” and “gratitude toward staff in general.” “Other” included “consideration for the physical condition of staff members” and “well‐wishes from children who cannot visit the patient” (Table 1).

**TABLE 1:** Main contents of opinions

Content categories	Sub‐categories	Items	Example
Complaints	Facilities, goods, and medical care	Taste and format of hospital food	The food does not taste good. At first I thought it was perhaps because of the anticancer drug, but I hear many people say the same. Can you not make the food meet the regular hospital standards?
		Air conditioning (temperature, humidity, and ventilation) management	I think it would be better to put a humidifier in the rooms because I feel that the heating is sometimes too high. Furthermore, I think it would be good to establish a permanently cool room (resting place) that has a little air conditioning somewhere within the hospital.
			Maybe it's the smell of the injection solution, but the smell is very strong in the outpatient treatment room in 3F. I wish you'd use an air freshener or something. It makes me want to puke.
		Making appliances (mostly TVs) within the ward free of charge	After being hospitalized, I felt that the TV card was too expensive.
	People	Responses and attitudes of nurses	Careless words cast by some of the ward nurses hurt me a lot. I wish they would be more considerate.
		Responses and attitudes of doctors	Because the patients are very worried, doctors talking to them when the endoscope is being inserted or during treatment could ease their distress. However, the doctor who assisted me did not talk to me at all during this time (was silent). I was in pain and felt nauseous, and it was very difficult for me.
		Responses and attitudes of unidentified staff members	Please address the elderly appropriately. Maybe they are acting like children because they are senile, but they are seniors in life. If my parents were treated the way I saw one elderly person being treated, I would feel really sad.
	Time	Waiting time for outpatient consultations is long	The appointment time was never honored. Perhaps too many bookings are made? This makes booking appointments meaningless.
		Waiting time in pharmacy is too long	I felt sick because the waiting time in the pharmacy was too long.
Compliments	Facilities, goods, and medical care	Environment (e.g., cleanliness, quietness, seasonal characteristics, expansiveness, great view, no smells associated with hospitals, etc.)	The hospital was hygienic and impeccably cleaned
			The hospital is like a hotel. The view from the 11th floor is great.
		Facilities (e.g., latest medical equipment, library, kids room, computers, bed‐side terminals, etc.)	The kids room and restaurant, patient library, were great.
			I felt truly relaxed when listening to the piano music playing in the hall. It was a moment in which I felt truly at ease.
	People	Gratitude toward staff in general	I was able to have a pleasant inpatient experience because all my interactions with the doctors, nurses, meal staff, and window cleaners were great.
		Gratitude toward doctors and nurses	The doctors, nurses, and all the other staff in the ward were very kind. They were all considerate toward patients and their families. When they communicated with us, they were always using kind words with a smile. I am truly grateful for this. Although I kept bothering them because there were so many things I did not now, I feel truly glad that I was able to be hospitalized in this hospital.
		Appreciation of reception staff and attitude of the nurses	Because all of the nurses spoke to me in a friendly manner, I was able to have an enjoyable hospital experience. Patients who come from afar, myself included, are not necessarily able to always see their families and friends, so the nurses became a huge source of support.
Other	Consideration for the physical condition of staff members		I think it was a hospital staff member, but at the vending machine, I saw a person who was providing support, standing at all times despite being pregnant. If possible, I want her to be able to sit down even at work.
	Well‐wishes from children who cannot visit the patient		Please help my grandmother because I want her to get well soon.

Regarding the target sub‐categories of complaints and compliments, the breakdown of complaints revealed 1263 complaints (53.4%) regarding “facilities, goods, and medical care,” 915 (38.7%) regarding “people,” 181 (7.7%) related to “time,” and four (0.2%) classed as “other.” Regarding compliments, 183 (17.6%) related to “facilities, goods, and medical care,” 859 (82.4%) related to “people,” and 0 related to “time” and “other.”

When opinions in the “people” subcategory were divided between “institution staff” and “non‐institution individuals,” there were 776 (84.8%) and 139 (15.2%) complaints, respectively, whereas all compliments were related to “institution staff” (859, 100%).

### 3.3 Responses to complaints

The classification of SCC's responses to complaints yielded 1003 cases (42.4%) of “responded,” 338 cases (14.3%) of “not responded,” and 1022 cases (43.3%) of “difficult to respond.” Of the complaints, primary examples of “responded” included “the Washlet (i.e., a toilet with a bidet function) is out of order,” “the windows are dirty,” and “I want you to make hand soap available.” Examples of “did not respond” included requests for “free parking” and “entertainment‐related objects.” Examples of “difficult to respond” included “the waiting time is long” and “dissatisfaction with hospital food.” Concerning complaints regarding “people,” compared with other methods of opinion gathering, opinions obtained via suggestion boxes contained unclear details concerning “institution staff.” For this reason, many such opinions were classified as “difficult to respond,” as no concrete answer could be provided (Table 2).

**TABLE 2:** Main contents of response

Response categories	Example
Responded	**Equipment failure** When I checked the computer in the day room of the ward, I found that it was out of order and immediately repaired it. **Shortage of goods** The patient library purchases about 30 general books and medical books every month. We will inform you about new books on the bulletin board in the library, so please check there.
No need to respond	**Parking fees** The fee for the outpatient carpark is paid by the user to cover the cost of managing the carpark and for the efficient use of the limited parking spaces. Free use for under 30 minu in the outpatient carpark is assumed to be used by the patient's transportation vehicle; otherwise it is assumed that the carpark will be used for between 30 min and 4 h, at a cost of 100 yen. Please understand that the fee is not high compared with neighboring hospital carparks. For those who have permission to accompany you, an exemption will apply if they park for more than 8 h, and the cost will be 200 yen a day for up to 24 h. Please contact the ward staff if you wish to use this exemption. **Setting up entertainment‐related objects** We are not considering preparing Othello, which is not always necessary in medical treatment. If you wish, please prepare your own.
Difficult to respond	**Waiting times** The waiting time in outpatient clinics is an issue for the entire hospital, and efforts are being made to shorten waiting times. However, long waiting times may still occur depending on the type of medical treatment. We will continue to attempt to reduce waiting times for medical examinations, so we hope you will understand. **Details unknown** Regarding an opinion about a nurse, we have confirmed it with the person in charge of the department as an anonymous opinion. However, we could not give individual guidance to the staff member who was the subject of the opinion. We instead decided to disseminate the content of the opinion through meetings and other occasions to alert the staff.

### 3.4 Temporal changes

**FIGURE 3:**
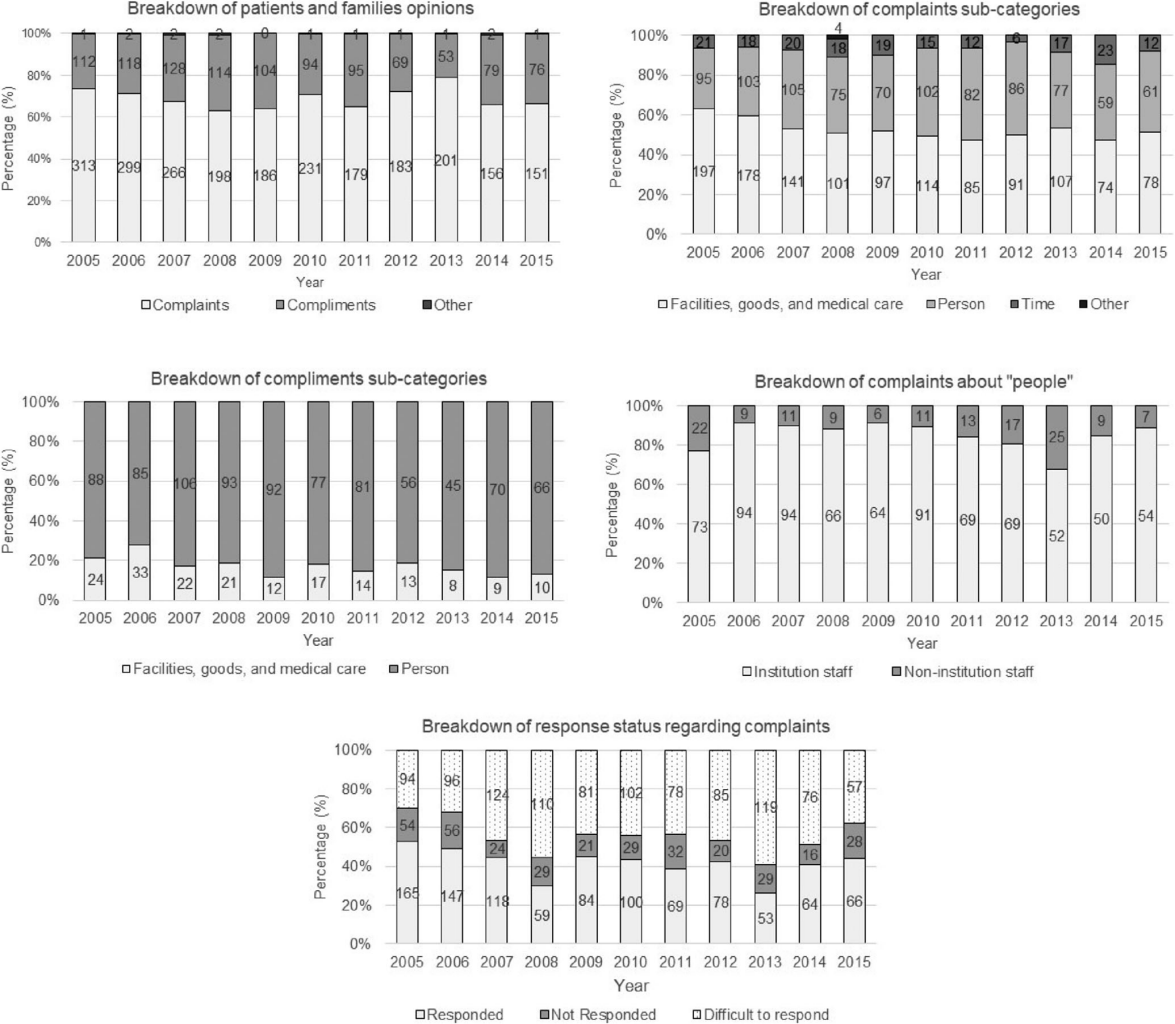
Change over time in content categories and sub‐categories of patients' and families' opinions and response status regarding complaints

Figure 3 shows the percentage for each content category of opinions of patients and families for each year. Although the number of opinions decreased annually, the ratio of complaints to compliments remained generally consistent over time, with complaints at 70% and compliments at 30%.

In the target sub‐categories of complaints and compliments, the number of complaints about “facilities, goods, and medical care” gradually decreased from 60% to 50% over time, while those related to “people” increased from 30% to 40%, indicating a gradual increase over time (Figure 3). Complaints about “time” were consistently recorded at around 10%. Conversely, while there were slight fluctuations over time concerning the target sub‐categories of compliments, those related to “people” accounted for approximately 80%.

For complaints regarding “people,” “institution staff” accounted for 80–90% of these complaints over the studied period, with most concerning doctors and nurses. Complaints about “non‐institution individuals” were mostly related to the manners of other patients and their families. Compliments were always directed to facility staff.

Changes over time with regard to facilities' responses to complaints were in the range of 40–50% for “responded,” 10–20% for “did not respond,” and 30–50% for “difficult to respond” (Figure 3).

## 4 DISCUSSION

To realize the paradigm shift in cancer treatment from medical treatment for curing a disease to medical care that includes both curing and caring for patients, it is necessary to provide care to patients and their families besides the treatment of cancer lesions. To this end, it is necessary to provide holistic medical care built upon a relationship of trust between patients and families and staff within medical institutions. As part of this effort, SCC emphasizes honest dialog between patients, families, and staff. SCC actively seeks the opinions of patients and families to learn from them, and responds to received opinions.

Of the various methods used for collecting opinions, submission to suggestion boxes includes both identifiable and anonymous submissions. Therefore, compared with other methods of opinion gathering that require indicating the identity of the person expressing the opinion, patients and families can express their opinions via suggestion boxes to or about medical institutions and employees without reservation.^17^ However, because one‐sided opinions based on erroneous understandings by patients and families can be submitted to these boxes,^17^ SCC applies clear and established response policies after carefully investigating the facts of each submission.

Of the 3419 opinions reviewed in this study, the ratio of complaints to compliments was 7:3. Previous studies in Japan^18^, ^19^, ^20^ reported that, while the number of submissions varied depending on the number of beds and length of periods studied, the ratio of complaints to requests/gratitude generally ranged between 6:4 and 8:2, which is a similar trend to that observed in the present study.

Regarding the sub‐categories of complaints, “facilities, goods, and medical care” accounted for 53.4%; “people” accounted for 38.7%, “time” accounted for 7.7%, and “other” accounted for 0.2%. Regarding the facilities' responses to complaints, “responded” accounted for 42.4%, which included cases of aging/breakdown of facilities and equipment, insufficient cleaning, and shortage of goods. Regarding medical facilities in general, it is common for attention to be paid to medical care management without considering patients' daily needs. From this perspective, the opinions of patients and families were useful for SCC's Management Division to identify equipment failure and shortage of goods, which could only be done via patients and their families. SCC could then utilize these insights to improve the quality of facilities.

“No need to respond” accounted for 14.3% of cases, with complaints including requests for “making paid parking free” and “setting up entertainment‐related objects.” In addition, it was found that some patients and their families did not necessarily recognize that hospitals are inherently places for group living, and that there are natural limitations to the extent to which SCC can improve the quality of life for individuals. For complaints of this nature, SCC considers it important to not respond to such opinions/suggestions, but to resolutely appeal to patients and their families to protect and adhere to the group living conditions at the hospital, as such responses can help improve the quality of individual medical care as well as the overall quality of the hospital.

Complaints classed as “difficult to respond” accounted for 43.3%. While some of these complaints were justified, others included matters that could not be addressed because SCC would require more staff or a considerably larger budget, or because the content was unclear. In addition, some of these complaints stated that the waiting time for consultations was taxing. However, these complaints had various causes, with no feasible solution that can be adopted by SCC to address all cases.

Complaints regarding “people” also accounted for a considerable number of opinions. However, investigation into these complaints was often impossible, because the items in relation to the person against whom the complaint was lodged and/or the facts related to the issue were unclear. In such cases, the content of the submission was still presented to the relevant parties, and they were encouraged to acknowledge the feelings of patients and their families. In this way, staffs were encouraged to learn about the importance of honest dialog.

Furthermore, regarding complaints about “people,” from the other opinion‐gathering methods implemented by SCC, reports from both medical staff and Patient Support & Inquires involved patients and families directing their complaints to the person in charge, with the content of their complaints being very specific. Using such methods can enable effective investigations into complaints and appropriate measures to address the situations (e.g., providing guidance to staff members). These findings suggest the clear necessity of using various information‐gathering measures concurrently, to best respond to complaints.

Cases related to “people” accounted for 80% of compliments, all of which were directed to staff affiliated with the institution. The suggestion box was used as a method for patients and their families to express their gratitude and appreciation. Mattarozzi et al.^21^ reported that “not only complaints, but also instances of praise, are a potentially important source of information regarding health care aspects that play a role in patient satisfaction and quality of care.” Compliments can be utilized to promote effective medical care that emphasizes patients' perspectives. In addition, compliments are thought to effectively motivate staff members and/or related departments toward task completion. For this reason, at SCC, staff and/or related departments that receive compliments from patients and families are notified accordingly.

The temporal changes in the number of opinions identified in this study showed a decreasing trend during the 11‐year period. Over this time, the total number of out‐ and in‐patients at SCC increased 1.4 times, and the proportion of opinions based on the total number patients halved. It is speculated that one factor behind this decreasing trend is the institution's responses to the opinions of patients and their families. However, while the number of complaints decreased according to the opinion‐gathering system, the number has not drastically declined. This has led to the conclusion that it is necessary to devise methods to address complaints labeled as “difficult to respond,” particularly when such complaints are justified. It is also necessary for SCC to continue appealing to patients and their families regarding the importance of adhering to group‐living best practices while in the hospital. There are three major limitations in this study that could be addressed in future research. The study focused on a single Japanese cancer center and used a secondary analysis of existing data. Furthermore, the classification method used in this study was developed by one researcher, with two researchers confirming its validity. As future issues arise in the classification method, we will evaluate its objectivity and the possibility of using it throughout Japan and overseas.

## 5 CONCLUSIONS

In this study, the opinions of patients and their families at SCC were analyzed using the booklet “*A Collection of Opinions*,” developed over an 11‐year period from 2005 to 2015. Through this analysis, we were able to report the characteristics and response status of these opinions submitted as suggestions. In particular, SCC was able to respond effectively to 40% of the complaints, which accounted for 70% of the total opinions. Among these complaints, there were many issues that were difficult for facility staff to notice, and, as such, they were useful in improving the hospital quality. In addition, compliments were an important source of information for improving patient satisfaction and the quality of medical care. Compliments were also linked to improving the motivation of individuals and/or related departments to adequately perform their duties. Activities aimed at taking the opinions of patients and families into account were considered to be an important method for strengthening the trust relationship between healthcare professionals and patients and families. These activities were also deemed to be crucial steps in implementing holistic medical care practices.

## CONFLICT OF INTEREST

The authors have stated explicitly that there are no conflicts of interest in connection with this article.

## AUTHOR CONTRIBUTIONS

All authors had full access to the data in the study and take responsibility for the integrity of the data and the accuracy of the data analysis. Conceptualization, K.T.; Methodology, K.T.; Investigation, T.N., K.Y., A.N.; formal analysis, K.T.; data curation, K.T.; validation, K.T., K.Y., A.N.; Writing ‐ Original Draft, K.T., K.Y.; Writing ‐ Review & Editing, K.T., T.N., A.N., K.Y.; Visualization, K.T.; Supervision, K.Y.

## ETHICAL STATEMENT

This study was conducted with the approval of the SCC Ethics Review Committee (Number: T 28–36–28‐1) and we used secondary data relating to the year of the submissions, the opinions, and the responses, excluding personal information.

## Data Availability

Data sharing is not applicable in the present study.
